# 

**Published:** 1872-01

**Authors:** 


					﻿Three Dollars a Year.
Specimen Copies, 30 Cents.
Vol. XXIX.
January, 1872.
Number i.
THE
(Tltirana jlinlical J|tniimal.
J. ADAMS ALLEN, M.D., LL.D., WALTER HAY, M.D.,
EDITORS.
CHICAGO:
W. B. KEEN, COOKE & CO., Publishers,
No. 408 Wabash Avenue.
The postage on this Journal is 24. cents a year—payable quarterly in advance.
				

